# Mast cell regulation of Na-glutamine co-transporters B0AT1 in villus and SN2 in crypt cells during chronic intestinal inflammation

**DOI:** 10.1186/s12876-015-0275-5

**Published:** 2015-04-15

**Authors:** Soudamani Singh, Subha Arthur, Jamilur Talukder, Balasubramanian Palaniappan, Steven Coon, Uma Sundaram

**Affiliations:** 1 Department of Clinical and Translational Sciences, Joan C. Edwards School of Medicine, Marshall University, 1600 Hal Greer Blvd., Huntington, WV 25701 USA; 2 Department of Biology, LeMoyne-Owen College, Memphis, TN 38126 USA; 3 Boston University School of Medicine, Boston, MA 02118 USA

**Keywords:** Na-glutamine co-transport, B0AT1, SN2/SNAT5, Ketotifen and Mast cells

## Abstract

**Background:**

In the chronically inflamed rabbit small intestine, brush border membrane (BBM) Na-glutamine co-transport is inhibited in villus cells (mediated by B0AT1), while it is stimulated in crypt cells (mediated by SN2/SNAT5). How mast cells, known to be enhanced in the chronically inflamed intestine, may regulate B0AT1 in villus and SN2/SNAT5 in crypt cell is unknown. Thus, the aim of the present study is to determine the regulation of B0AT1 and SN2/SNAT5 by mast cells during chronic enteritis.

**Methods:**

Chronic intestinal inflammation was induced in male rabbits with intra-gastric inoculation of *Eimeria magna* oocytes. Rabbits with chronic inflammation were treated with ketotifen (10 mg/day) or saline (Placebo) for 2 days. Villus and crypts cells were isolated from the rabbit intestine using the Ca++ chelation technique. Na/K-ATPase activity was measured as Pi from cellular homogenate. BBM vesicles (BBMV) were prepared from villus and crypt cells and uptake studies were performed using rapid filtration technique with ^3^H-Glutamine. Western blot analyses were done using B0AT1 and SN2 specific antibodies.

**Results:**

In villus cells, Na-glutamine co-transport inhibition observed during inflammation was completely reversed by ketotifen, a mast cell stabilizer. In contrast, in crypt cells, Na-glutamine co-transport stimulation was reversed to normal levels by ketotifen. Kinetic studies demonstrated that ketotifen reversed the inhibition of B0AT1 in villus cells by restoring co-transporter numbers in the BBM, whereas the stimulation of SN2/SNAT5 in crypts cells was reversed secondary to restoration of affinity of the co-transporter. Western blot analysis showed that ketotifen restored immune-reactive levels of B0AT1 in villus cells, while SN2/SNAT5 levels from crypts cell remained unchanged.

**Conclusion:**

In the present study we demonstrate that mast cells likely function as a common upstream immune pathway regulator of the Na-dependent glutamine co-transporters, B0AT1 in villus cells and SN2 in crypts cells that are uniquely altered in the chronically inflamed small intestine.

## Background

Glutamine, the most abundant amino acid plays a vital role in the regulation of cell specific processes including metabolism (e.g., oxidative fuel, gluconeogenic precursor, and lipogenic precursor), cell integrity (apoptosis, cell proliferation), protein synthesis and degradation, contractile protein mass, redox potential, respiratory burst, insulin resistance, insulin secretion, and extracellular matrix synthesis [1-5]. In addition, glutamine is not only the primary source of energy for intestinal cells, but has also been shown to play an important role in maintaining mucosal health and integrity during gastrointestinal disorders [3,6].

As the principal nutrient for the intestinal epithelium, glutamine is primarily assimilated through Na-dependent glutamine co-transporters expressed on the brush border membrane (BBM) of enterocytes in rabbit small intestine [7,8]. Previously we also demonstrated that Na-glutamine co-transport is predominantly mediated by B0AT1 (SLC6A19) [7] in villus cells while it is mediated by SN2/SNAT5 (SLC38A5) in crypt cells [8]. Our studies have also shown extensively the functional and molecular properties of these transporters, including substrate specificity, kinetic properties, and protein expression. Based on our observations, we suggested that glutamine uptake was approximately four times greater in villus cells as compared to crypt cells, standardized for BBM protein of these cells [7,8]. Further, in the chronically inflamed rabbit small intestine we demonstrated that B0AT1 was inhibited in villus cells while SN2/SNAT5 was stimulated in crypt cells [9]. Furthermore, we showed that the mechanism of inhibition of B0AT1 was secondary to a reduction in the number of BBM co-transporters whereas the stimulation of SN2/SNAT5 was secondary to enhanced affinity of the co-transporter for glutamine.

Over the years there has been increasing awareness in the pathophysiology of inflammatory bowel disease (IBD) and the role of various mucosal leukocytes, such as mast cells, in the pathogenesis of the disease. A marked increase in the number of mast cells accompanied by elevated degranulation of mucosal mast cell chemical mediators and cytokines such as histamine, substance P and tumor necrosis factor α have been observed in the mucosa of the ileum and colon of patients with IBD [10-13]. Also, regulation of mast cell activation by mast cell stabilizing agents has been shown to reduce inflammation and fibrosis of intestinal lesions of the experimental colitis [14]. However, what is not clear is whether mucosal mast cells may affect Na-glutamine co-transport during intestinal inflammation.

The wide variety of immune-inflammatory mediators known to be endogenously produced in the chronically inflamed ileum may, at least in part, have an effect on nutrient transport pathways including Na-glucose, Na-alanine and Na-taurocholate co-transport [15-18]. Nevertheless, it is not known whether a given immune-inflammatory mediator pathway may be responsible for Na-glutamine co-transport alterations during chronic ileitis, but glucocorticoids, a broad-spectrum immune modulator used for the treatment of IBD, has been shown to alleviate the alterations. We have shown that methylprednisolone reversed the inhibition of B0AT1 in villus and stimulation of SN2/SNAT5 in crypt cells during chronic intestine inflammation [19]. These observations suggest that the alterations in Na-glutamine co-transport are actively regulated by immune-inflammatory mediators in the chronically inflamed intestine. However, it is not known if mast cell mediators may regulate Na-glutamine co-transport activity during chronic enteritis. Thus, the aim of the present study was to assess whether stabilization of mast cell membrane by ketotifen will alleviate the alterations in B0AT1 and/or SN2/SNAT5 during chronic enteritis.

## Methods

### Animals: induction of chronic inflammation, drug treatment and cell isolation

Pathogen free New Zealand White male rabbits (Charles River Laboratories International Inc.) were used for the study. Four cohorts of animals, 4 animals in each cohort, were used: normal, inflamed, normal + ketotifen and inflamed + ketotifen. Chronic intestinal inflammation was induced in rabbits by intragastrically inoculating them with *Eimeria magna* oocytes as previously reported [18,20]. Normal and inflamed rabbits that were injected intramuscularly with saline were used as untreated controls. For drug treatment, normal and inflamed rabbits were intramuscularly injected with ketotifen (10 mg/kg body weight), a noncompetitive H1-antihistamine and mast cell stabilizer, 12 and 13 days post coccidia inoculation and the animals were euthanized on day 14. All animal handling, treatments and euthanization were carried out according to the protocol approved by the Institutional Animal Care and Use Committee of West Virginia University (ACUC protocol # 12–0102).

Villus and crypt cells were isolated from the rabbit ileum by a calcium chelation technique as previously described [18]. Briefly, a 3-ft section of ileum was filled and incubated with cell isolation buffer (0.15 mM EDTA, 112 mM NaCl, 25 mM NaHCO_3_, 2.4 mM K_2_HPO_4_, 0.4 mM KH_2_PO_4_, 2.5 mM L-glutamine, 0.5 mM β-hydroxybutyrate, and 0.5 mM dithiothreitol; gassed with 95% O_2_ and 5% CO_2_, pH 7.4, at 37°C) for 3 min and gently palpitated for another 3 min to facilitate cell dispersion. The buffer was then drained out from the ileal section, phenylmethylsulfonyl fluoride was added, and the suspension was centrifuged at 100 g for 3 min. Cells to be used for BBM vesicle (BBMV) preparation were frozen immediately in liquid nitrogen and stored at −80°C until required.

### β-Hexosaminidase assay

When activated mast cells undergo degranulation they release a substantial amount of enzymes that mediate several inflammatory pathways. One such enzyme that is released is β-Hexosaminidase, the levels of which are estimated as an index of mast cell degranulation during inflammation. In the present study, β-Hexosaminidase assay was performed as previously reported [21], to detect mast cell degranulation *in vivo*. Briefly, enterocytes lysed with 1% Triton X-100 were incubated with the substrate solution (P-nitrophenyl-Nacetyl-β-D-glucosaminide from Sigma, St Louis, MO) in 0.1 M citrate buffer (pH 4.5) for 60 min at 37°C. The reaction was terminated by the addition of 0.2 M NaOH/0.2 M glycine. Using an enzyme-linked immunosorbent assay reader, absorbance was acquired at 405 nm and the results were expressed as percentage β-Hexosaminidase activity relative to control.

### Na-K-ATPase measurement

Na-K-ATPase was measured as Pi liberated [22] in cellular homogenates from the same amount of cells from all experimental conditions as previously described [9,18,22]. Enzyme-specific activity was expressed as nanomoles of Pi released per milligram protein per minute.

### Uptake studies in villus and crypt cells

Intact villus and crypt cell uptakes were done using previously described protocol [9,18]. Briefly, villus or crypt cells (100 mg wet wt.) were washed and resuspended in HEPES buffer containing 0.2 mM glutamine, 4.5 mM KCl, 1.2 mM KH_2_PO_4_, 1.0 mM MgSO_4_, 1.25 mM CaCl_2_, 20 mM HEPES, and either 130 mM sodium chloride or choline chloride and was gassed with 100% O_2_ (pH 7.4 at 37°C). Ten μCi of ^3^H-glutamine was added to 1 mL of cell suspension in HEPES buffer and 100 μL aliquots were removed at 2 minutes and mixed with 1 mL ice-cold stop solution (choline-HEPES buffer) to stop the uptake. The mixture was then filtered on 0.65 μm Millipore (Bedford, MA; HAWP) filters and washed twice with ice cold-stop solution. The filter was dissolved in 5 mL Ecoscint and the radioactivity was determined in a Beckman Coulter LS6500 Scintillation counter.

### BBMV preparation and uptake studies

BBMV from rabbit ileal villus and crypt cells were prepared by CaCl_2_ precipitation and differential centrifugation as previously described [18]. Uptake studies were performed by rapid filtration technique [18,20]. Briefly, 5 μL of BBMV resuspended in vesicle medium (100 mM choline chloride, 0.10 mM MgSO_4_, 50 mM HEPES-Tris (pH 7.5), 50 mM mannitol, 50 mM KCl) were voltage clamped with 10 μM valinomycin and 100 mM carbonyl cyanide p-(tri-fluoromethoxy) phenyl-hydrozone. The vesicles were then incubated in 95 μL of reaction medium (50 mM HEPES-Tris buffer (pH 7.5), 0.2 mM glutamine, 20 μCi ^3^H-glutamine, 0.10 mM MgSO_4_, 50 mM KCl, 50 mM mannitol, 100 mM of either NaCl or choline chloride) and at desired time points uptake was arrested by mixing with ice-cold stop solution of 50 mM HEPES-Tris buffer (pH 7.5), 0.10 mM MgSO_4_, 75 mM KCl, and 100 mM choline chloride. The stopped reaction mixture was filtered on a 0.45 μm Millipore (HAWP) filter and washed with 10 mL ice-cold stop solution, twice. Filters were then dissolved in Ecoscint solution and radioactivity was determined in a Beckman Coulter LS6500 Scintillation counter.

### Kinetic studies

Na-dependent glutamine uptake was performed as described above in BBMV from all experimental conditions. Kinetic parameters were derived from Na-dependent glutamine uptake at 6 seconds performed at varying concentrations of extra vesicular glutamine (0.2, 0.5, 1, 5, 10, 25, 50, 75 and 100 mM). Uptake values were analyzed for simple Michaelis-Menten kinetics using a non-linear regression data analysis using GraphPad Prism 4 (San Diego, CA).

### Western blot analysis

BBM protein was used for all Western blot experiments. BBM was solubilized in a buffer consisting of 50 mM Tris–HCl (pH 7.4), 1% Igepal, 150 mM NaCl, 1 mM EDTA, 1 mM PMSF, 1 mM Na_3_VO_4_, 1 mM NaF, supplemented with a mixture of a protease inhibitor (Sigma, St.Louis, MO). Equal amounts of protein were denatured in a sodium dodecyl sulfate (SDS)/sample buffer (20 mM Tris pH 7, 12% glycerol, 2% SDS, 0.01% bromophenol blue, and freshly added 1 mM Dithiothreitol) and separated by electrophoresis on a 4%–20% gradient Gel (Bio-Rad Laboratories, Hercules, CA). Proteins on the gel were transferred to a polyvinylidene membrane which was blocked with 5% dry milk in TBS (20 mM Tris, pH 7.5, 150 mM NaCl) with 0.1% Tween-20 and then incubated with a goat polyclonal antibody against B0AT1, overnight at 4°C followed by incubation with an anti-goat IgG conjugated to horseradish peroxidase (Jackson Immunoresearch Laboratories, West Grove, PA) for an hour at room temperature. For SN2/SNAT5, a chicken polyclonal antibody against SN2/SNAT5 was used as the primary antibody and an anti-chicken IgG was used as the secondary. Both the primary antibodies were obtained through the custom antibody services provided by Invitrogen. ECL Western blotting detection reagent (GE Healthcare Bio-Sciences, Piscataway, NJ) was used to detect the immobilized protein. The resultant chemiluminescence was detected using biomax film (Kodak, Rochester, NY) and the intensity of the bands was analyzed by a FluorChem™ instrument (Alpha Innotech, San Leandro, CA).

### Statistical analysis

Data are shown as mean ± SEM in all figures. All individual uptakes were done in triplicate. The ‘n’ number for any set of uptake experiments or Western blot analysis refers to vesicle or isolated cell preparations from different animals. Data were analyzed using one-way analysis of variance (ANOVA) to assess the significance between control and experimental groups by using GraphPad Instat 4 (San Diego, CA) for statistical analysis and a *p* value of less than 0.01 was considered significant.

## Results

### Effects of ketotifen on mast cell β-hexosaminidase in intestinal mucosa

**Figure 1:**
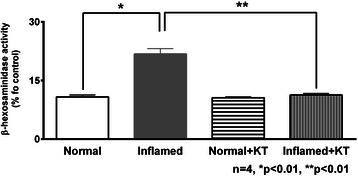
Effects of ketotifen on β-hexosaminidase activity in enterocytes. β-hexosaminidase activity normalized to 1 in enterocytes and expressed as % relative to control. β-hexosaminidase activity was significantly increased in enterocytes from the chronically inflamed intestine when compared to controls. Ketotifen treatment restored β-hexosaminidase activity during chronic enteritis while having no effect in the normal intestine.

β-hexosaminidase activity was significantly increased in enterocytes from the chronically inflamed intestine indicating enhanced degranulation of mast cells. Whereas, in enterocytes from ketotifen treated animals with chronic enteritis, β-hexosaminidase release returned to near normal levels indicating that mast cell degranulation was prevented (Figure 1).

### Effect of ketotifen on Na-glutamine co-transport in intact villus and crypt cells

**Figure 2:**
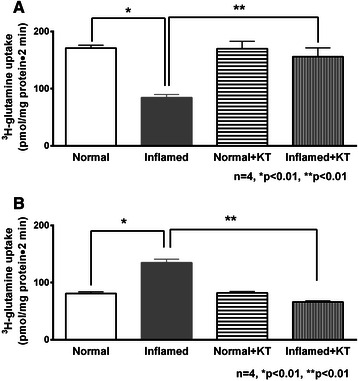
Effect of ketotifen on Na-glutamine co-transport in intact cells. **A**, Villus cells. Na-dependent glutamine uptake was determined as a function of ^3^H-glutamine uptake in the presence of extracellular Na minus uptake in the absence of extracellular Na. Na-dependent glutamine uptake was significantly decreased in villus cells from the chronically inflamed intestine and treatment with ketotifen reversed this inhibition. Ketotifen had no effect on Na-glutamine co-transport in villus cells from the normal intestine. **B**, Crypt cells. Na-glutamine co-transport uptake was significantly increased in crypt cells during chronic intestinal inflammation and treatment with ketotifen reversed this stimulation. Ketotifen had no effect on Na-glutamine co-transport in crypt cells from the normal intestine.

Na-glutamine co-transport, which is known to be significantly inhibited in the villus cells during chronic intestinal inflammation was completely reversed by ketotifen treatment (Figure 2A, 84.4 ± 5.3 pmol/mg protein•2 min in villus cells from inflamed and 156 ± 15.4 from ketotifen + inflamed). Ketotifen did not have any effect in villus cells from the normal intestine (Figure 2A, 171 ± 5 pmol/mg protein•2 min in normal and 170 ± 12.8 in ketotifen treated normal villus cells).

In contrast, in crypt cells, Na-glutamine co-transport which was stimulated during chronic intestinal inflammation was reversed to near normal levels by ketotifen treatment (Figure 2B, 135 ± 6 pmol/mg protein•2 min in crypts from inflamed intestine and 66 ± 2 in inflamed + ketotifen). Here again, ketotifen did not affect Na-glutamine co-transport in crypt cells isolated from normal animals treated with ketotifen (Figure 2B, 81 ± 3 pmol/mg protein•2 min in normal and 82.3 ± 2.6 in normal + ketotifen).

This data shows that ketotifen reverses the inhibition of Na-glutamine co-transport in villus cells and its stimulation in crypt cells to their normal levels during chronic enteritis.

### Effect of ketotifen on Na-K-ATPase activity in villus and crypt cells

**Figure 3:**
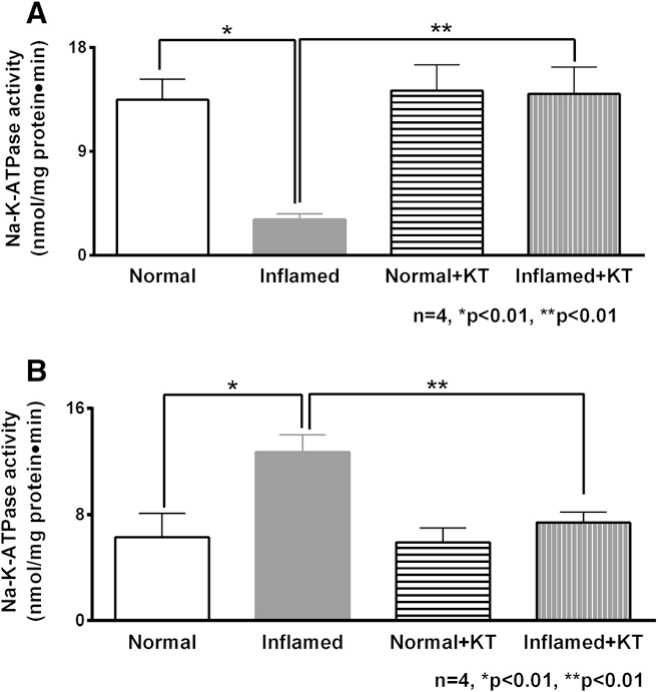
Effect of ketotifen on Na-K-ATPase activity. **A**, Villus cells. Na-K-ATPase activity that was significantly reduced in the villus cells during chronic enteritis was reversed by ketotifen treatment. **B**, Crypt cells. Na-K-ATPase activity was significantly elevated in the crypt cells from the chronically inflamed intestine and this stimulation was reversed by ketotifen treatment. Na-K-ATPase activity was unaffected in the crypt cells from normal intestine.

Basolateral membrane Na-K-ATPase provides the transcellular Na^+^ gradient required to drive Na-coupled transporters in the BBM. In villus cells from the chronically inflamed intestine, Na-K-ATPase activity was significantly inhibited and this inhibition was reversed by treatment with ketotifen (Figure 3A, 13.5 ± 1.8 nmol/mg protein•min in normal; 3.1 ± 0.5 in inflamed; 14.3 ± 2.2 in normal + ketotifen and 14 ± 2.3 in inflamed ± ketotifen). In contrast, in crypt cells Na-K-ATPase activity was significantly increased in the inflamed intestine, but this was also reversed by ketotifen (Figure 3B, 6.3 ± 1.8 nmol/mg protein•min in normal; 12.7 ± 1.3 in inflamed; 5.9 ± 1.1 in normal + ketotifen and 7.4 ± 0.8 in inflamed + ketotifen).

### Effect of ketotifen on Na-glutamine co-transport in villus and crypt cell BBMV

**Figure 4:**
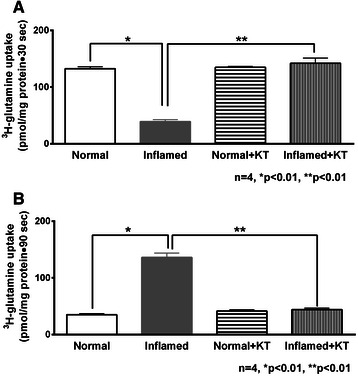
Effect of ketotifen on Na-glutamine co-transport in BBMV. **A**, Villus cells. Na-dependent glutamine uptake was significantly decreased in villus cell BBMV from the chronically inflamed intestine and this inhibition was reversed with ketotifen treatment. **B**, Crypt cells. Na-dependent glutamine uptake was significantly increased in crypt cell BBMV during chronic intestinal inflammation and this stimulation was reversed by ketotifen treatment.

While changes in Na-K-ATPase activity might be partially responsible for co-transporter alterations at the cellular level, it is important to determine whether mast cell mediators have any effect at the level of the co-transporters in the BBM. Ketotifen treatment of rabbits with chronic intestinal inflammation resulted in a significant reversal of Na-glutamine co-transport uptake in villus cell BBMV (Figure 4A, Na-glutamine co-transport in villus BBMV: in normal 133 ± 3.5 pmol/mg protein•30 sec, in chronically inflamed 39.1 ± 3.6, in normal + ketotifen 135 ± 1.7 and in inflamed + ketotifen 142 ± 9.2). It also reversed the stimulation of Na-glutamine co-transport in crypt cell BBMV (Figure 4B, Na-glutamine co-transport in crypt BBMV: in normal 34.3 ± 2 pmol/mg protein•90 sec, in chronically inflamed 136 ± 7.7, normal + ketotifen 41.6 ± 2.5, and in inflamed + ketotifen 44 ± 2.6).

These results indicate that mast cell stabilization reversed the inhibition of Na-glutamine co-transport in villus cell BBMV, and stimulation in crypts cell BBMV at the level of the BBM co-transporter.

### Kinetic studies

To determine the mechanism of alterations of the BBM glutamine co-transporters by ketotifen in inflamed villus and crypt cells, we performed BBMV kinetic studies. As the concentration of extra-vesicular glutamine was increased, glutamine uptake was stimulated and subsequently became saturated in all conditions. Kinetic parameters showed that ketotifen reversed the inhibition of B0AT1 in villus cells by restoring the *V*_*max*_ without affecting the affinity of the co-transporter (Table 1; *V*_*max*_ for glutamine uptake in BBMV was 3.4 ± 0.1 pmol/mg protein•6 sec in normal, 1.7 ± 0.03 in inflamed and 3.2 ± 0.1 in inflamed + ketotifen).

**Table 1:** **Kinetic parameters of Na-glutamine co-transport in villus cell BBMV**

	*V*_*max*_(nmol/mg protein•6 sec)	*K*_*m*_(mM)
**Control**	**3.4 ± 0.1**	**24 ± 1.5**
**Inflamed**	1.7 ± 0.03*	**27.1 ± 1.1**
**Inflamed + ketotifen**	3.2 ± 0.1*	**23.7 ± 1.9**

In villus cells, the maximal rate of uptake (*V*_*max*_) of Na-glutamine co-transport which was significantly diminished in chronically inflamed intestine was reversed by ketotifen treatment (n = 3, *p < 0.01).

In contrast, kinetic studies showed that ketotifen reversed the affinity of SN2/SNAT5, which was increased during chronic inflammation, without altering the maximal rate of uptake of glutamine (Table 2; *K*_*m*_ for glutamine uptake in crypt BBMV was 49.6 ± 4.8 mM in normal, 21.2 ± 0.4.3 in inflamed and 45 ± 0.6 in inflamed + ketotifen).

**Table 2:** **Kinetic parameters of Na-glutamine co-transport in crypt cell BBMV**

	*V*_*max*_(nmol/mg protein•6 sec)	*K*_*m*_(mM)
**Control**	**2.4 ± 0.9**	**49.6 ± 4.8**
**Inflamed**	**2.6 ± 0.4**	21.2 ± 4.3*
**Inflamed + ketotifen**	**2.3 ± 0.6**	45 ± 0.6*

In crypt cells, the affinity (*K*_*m*_) of Na-glutamine co-transport which was increased significantly during chronic intestinal inflammation was reversed back to its normal levels by ketotifen treatment (n = 3, *p < 0.01).

Thus, this data demonstrated that the mechanism of ketotifen mediated reversal of inhibition of B0AT1 activity in the villus cells from the chronically inflamed intestine was by the restoration of the number of co-transporters in the BBM. Whereas, the reversal of stimulation of SN2/SNAT5 activity in crypt cells by ketotifen was secondary to the restoration of the affinity of the co-transporter for its substrate.

### Western blot analysis

**Figure 5:**
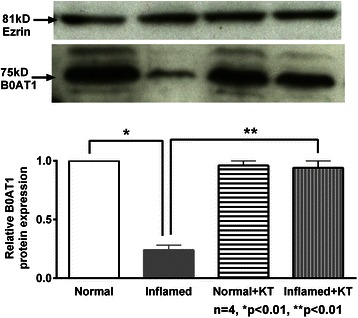
B0AT1 protein expression in villus cell BBM. A representative Western blot of BBM B0AT1 protein levels is shown in the upper panel. In the lower panel densitometric quantitation is shown. The relative protein expression of B0AT1 that was reduced during chronic intestinal inflammation was restored to near-normal levels by ketotifen treatment and remained unaffected in the normal intestine.

**Figure 6:**
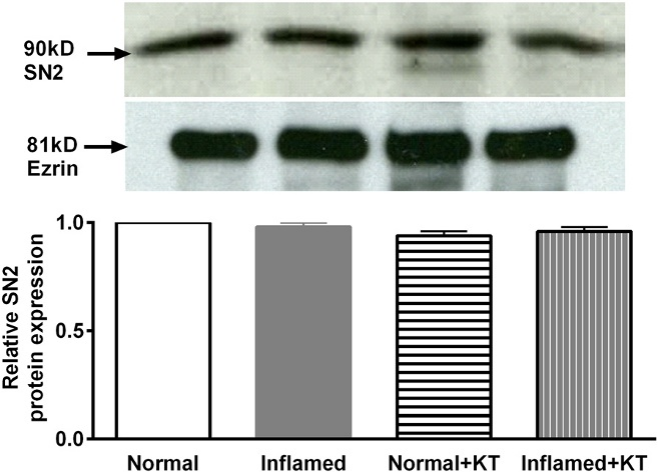
SN2/SNAT5 protein expression in crypt cell BBM. A representative Western blot of BBM SN2/SNAT5 protein levels is shown in the upper panel. In the lower panel densitometric quantitation is shown. The levels of BBM SN2/SNAT5 protein expression remained unaltered in all experimental conditions (n = 4).

Immunoreactive protein levels of B0AT1 and SN2/SNAT5 were determined in the villus and crypt cell BBM for all experimental conditions. Figure 5 shows a representative immunoblot of B0AT1 from villus cell BBM. Densitometric analysis of relative protein expression revealed that ketotifen treatment restored B0AT1 immunoreactive levels in the villus cell BBM from the chronically inflamed intestine and did not have any effect on the protein levels in the normal villus BBM. Figure 6 is a representative immunoblot of crypt cell BBM SN2/SNAT5 from all experimental conditions. Densitometric quantitation showed that the relative protein expression of SN2/SNAT5 was unchanged in the BBM of crypt cells from all experimental conditions.

This data in conjunction with the kinetic analysis above demonstrates that the mechanism of ketotifen mediated reversal of Na-glutamine co-transport during chronic intestinal inflammation is through the restoration of the B0AT1 numbers in villus and affinity of SN2/SNAT5 in crypt cells.

## Discussion

It is well documented that glutamine is an important nutrient required for the normal growth and differentiation of intestinal enterocytes [23,24]. Therefore, glutamine becomes more important to help restore the mucosal integrity and function of the small intestinal mucosa in chronic pathophysiological conditions [23]. There are two different Na dependent co-transport pathways that enable glutamine absorption in the rabbit intestine, namely B0AT1 in villus and SN2/SNAT5 in crypt cells [7,8]. It has been shown previously that B0AT1 is inhibited in villus during chronic intestinal inflammation, whereas SN2/SNAT5 is stimulated in crypt cells [9] and treatment with a broad spectrum immune modulator reversed these alterations during chronic intestinal inflammation [19]. In the chronically inflamed intestine, mast cells have been shown to be important in the pathogenesis and progression of disease [14,25]. However, the regulation of Na-glutamine co-transporter alterations by mucosal mast cells during intestinal inflammation is not known. Therefore, the aim of this study was to investigate the role of the mucosal mast cells on Na-glutamine co-transport changes during chronic intestinal inflammation.

In the present study, mast cell stabilization by ketotifen reversed the inhibition of B0AT1 in intestinal villus during chronic intestinal inflammation and the mechanism of reversal of inhibition was secondary to restoration of diminished co-transporter numbers in the villus cell BBM. The mechanism of B0AT1 inhibition in the chronically inflamed intestine, secondary to a reduction in co-transporter numbers in the villus cell BBM, appears to be unique to this co-transporter. This is because other villus cell BBM Na-amino acid and di-peptide transporters are affected by a different mechanism during chronic intestinal inflammation. We have previously shown that Na-alanine co-transport (mediated by ATB0) in villus BBM of the rabbit small intestine is inhibited during chronic intestinal inflammation, not secondary to alteration in the number of BBM co-transporters, but secondary to a reduction in the affinity of the co-transporter for alanine [26]. Similarly, the proton dipeptide co-transporter (PEPT1) was also significantly diminished during chronic intestinal inflammation and the mechanism of this inhibition was again secondary to a reduction in the affinity of the co-transporter rather than an alteration in the number of co-transporters on the BBM of villus cells [18]. However, a different class of Na-nutrient co-transporters, specifically, Na-glucose co-transporter SGLT1, is inhibited during chronic intestinal inflammation by a reduction in the number of BBM co-transporters [27].

Unlike villus cells, ketotifen reversed the stimulation of SN2/SNAT5 in crypt cells and the mechanism of reversal was secondary to the restoration of the affinity (*K*_*m*_) of the co-transporter for glutamine. SN2/SNAT5 is the only Na-nutrient co-transporter that has been reported to be present in the BBM of crypt cells from the rabbit small intestine [8]. Numerous studies demonstrate that Na-glucose [18] Na-alanine [17], Na-bile acid co-transport [16] and PEPT1 [25] are present only in villus cells, but not crypt cells in the rabbit intestine [16-18]. Thus, presence of Na-glutamine co-transport in crypt cells further emphasizes the importance of this nutrient for the well-being of intestinal epithelial cells. Further, unlike all other Na-nutrient co-transport processes which are inhibited during chronic intestinal inflammation [16,18,28], only SN2/SNAT5 is stimulated so as to potentially compensate for the inhibition of Na-glutamine co-transport in villus cells [9]. Though the reversal of Na-K-ATPase activity in ketotifen treated inflamed villus and crypt might appear to be partially responsible for the reversal of B0AT1 and SN2 activities, BBMV vesicle kinetic studies and Western Blot analyses suggest that they are directly regulated by specific immune inflammatory mediators. The differential mechanism of regulation of Na-glutamine co-transport in crypt as compared to villus, specifically, altered affinity of SN2/SNAT5 during chronic intestinal inflammation suggests that a specific immune inflammatory mediator/pathway might be responsible for each Na-glutamine co-transport alteration [29]. Activated mast cells are known to generate and release abundant quantities of preformed mediators such as tryptase, histamine, serotonins, proteases, proteoglycans and cytokines such as tumor necrosis factor α (TNFα) [30,31]. TNFα has been shown to trigger multiple signaling pathways that regulate innumerable target proteins. More specifically, it has been shown to regulate several transcription factor proteins such as nuclear factor kappa B and activating protein 1 [32]. In the present study, alteration of the transcription factors by TNFα activated signaling pathways, either by phosphorylation or dephosphorylation, might be responsible for altered B0AT1 protein transcription. Mast cells also release newly formed eicosanoids such as leukotriene C4, prostaglandin (PG)D2, and leukotriene B4. Of these, PGD2 has been found to play a major role in the progression of intestinal inflammation and has been shown to activate G-protein coupled receptors DP1 and DP2 resulting in the activation of adenylate cyclase and subsequent increase in intracellular cAMP levels and subsequent activation of the cAMP mediated protein kinase A (PKA) pathway [33,34]. The PKA pathway might regulate B0AT1 in the inflamed intestine by altering the phosphorylation status of transcription factors that regulate B0AT1 transcription. Similarly, PKA mediated direct phosphorylation of SN2 protein might be responsible for its altered affinity during inflammation. Previously, our laboratory has shown that an immune inflammatory mediator such as Leukotriene D4 alters the affinity of a different BBM amino acid co-transporter, specifically, Na-alanine co-transporter, ASCT1, and that this alteration was due to secondary changes in ASCT1 phosphorylation levels mediated by protein kinase C pathway [29,35].

In the present study, since mast cell stabilization reversed the increased affinity of SN2/SNAT5, as well as the inhibition of B0AT1 in the chronically inflamed rabbit intestine, it is reasonable to postulate that mast cell mediators function as an upstream common immune modulator of Na-glutamine co-transport alterations. Future studies are in order to determine the downstream immune inflammatory pathway/s and the specific inflammatory mediator/s that might be responsible for the regulation of Na-glutamine co-transporters B0AT1 and SN2 during intestinal inflammation.

## Conclusion

In conclusion, the uniquely altered Na-dependent glutamine co-transporters, inhibition of B0AT1 in villus and stimulation of SN2/SNAT5 in crypt cells, are regulated back to normal by mast cell stabilization in the chronically inflamed small intestine. The mechanisms of ketotifen mediated restoration of function are the same mechanisms that resulted in their original alteration, specifically, diminished co-transporter numbers in villus and enhanced affinity for glutamine in crypt cells during chronic intestinal inflammation. These observations indicate that mast cells likely function as an upstream broad-spectrum immune modulator responsible for the unique regulation of B0AT1 in villus and SN2/SNAT5 in crypt cells during chronic intestinal inflammation.
